# Toward point-of-care and amplification-free detection of human cytomegalovirus using CRISPR-Cas12a

**DOI:** 10.1016/j.isci.2026.116494

**Published:** 2026-06-23

**Authors:** Kavish A.V. Kohabir, A.W.J. Rietveld, Lars O. Nooi, Roos E. Beijer, Jeanne E. van Dongen, Jasper Linthorst, Rob M.F. Wolthuis, Marcel Jonges, Matthijs R.A. Welkers, Loes I. Segerink, Erik A. Sistermans

**Affiliations:** 1 Department of Human Genetics, Amsterdam UMC, Location Vrije Universiteit, Amsterdam, the Netherlands; 2 Amsterdam Reproduction and Development Research Institute, Amsterdam, the Netherlands; 3 Cancer Center Amsterdam Research Institute, Amsterdam, the Netherlands; 4 BIOS Lab on a Chip Group, MESA Institute for Nanotechnology, Technical Medical Center, Max Planck Institute for Complex Fluid Dynamics, University of Twente, Enschede, the Netherlands; 5 Amsterdam Institute for Immunology and Infectious Diseases, Amsterdam, the Netherlands; 6 Department of Medical Microbiology and Infection Prevention, Amsterdam University Medical Centers, Amsterdam, the Netherlands

**Keywords:** Molecular biology, Microbiology, Bioengineering, Biomedical engineering, Biotechnology

## Abstract

Human cytomegalovirus (hCMV) is a herpesvirus that establishes lifelong latency in myeloid cells, posing health concerns particularly in fetal development and in immunocompromised individuals. Point-of-care (PoC) detection of hCMV DNA in liquid biopsies supports timely diagnosis and proper mitigation. However, ultra-low concentrations and high fragmentation rates, challenge primer-based preamplification methods. We present a proof-of-concept amplification-free CRISPR-based assay, exploiting the inherent specificity and signal-amplification of Cas12a and improving signal using a combinatorial approach. Optimizing Cas12a’s *trans*-cleavage activity and multiplexing hCMV loci, significantly increased detection sensitivity in-bulk. Additionally, we found that AsCas12a *trans*-cleaves cytosine-rich reporters 4× more efficiently than conventional probes, further improving assay kinetics to reach a femtomolar limit of detection. Translating these optimizations to a microfluidic assay enables sensitive detection even if additional measures may be needed for quantitative, single molecule measurements. Our assay opens avenues toward PoC detection in low-resource settings, supporting effective and affordable infection management.

## Highlights

• Multiplexing crRNAs helps Cas12a-based detection of low-concentration fragmented DNA
• Multidimensional buffer and probe tuning enables detection of femtomolar DNA in-bulk
• Kinetics of AsCas12a Ultra and LbCas12a are comparable under optimized reaction conditions
• Application in a microfluidic format demonstrates promise for amplification-free PoC testing

## Graphical abstract

**Figure:**
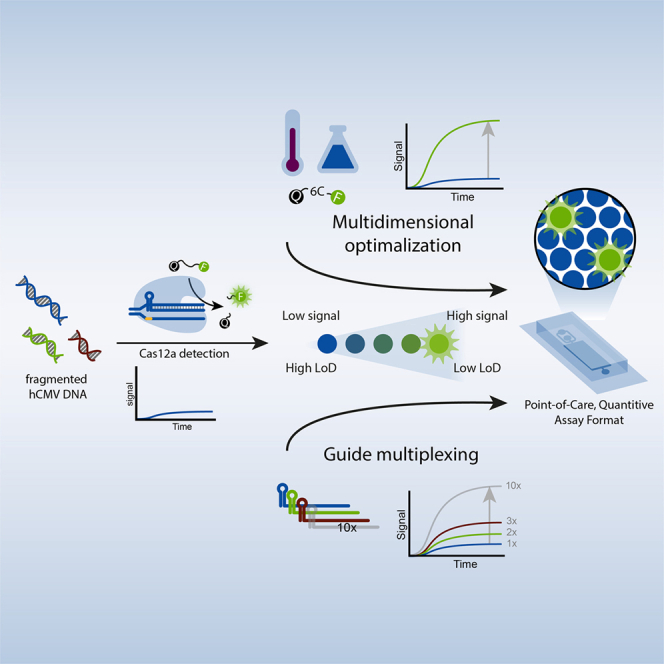

## Introduction

Human cytomegalovirus (hCMV), or human betaherpesvirus 5 (HHV5), is a herpesvirus that establishes lifelong latency in hematopoietic cells, but is typically controlled by the immune system upon clinical manifestation.^1^ hCMV can reactivate through various stimuli, but exact mechanisms remain to be resolved.^2^ Particularly in transplant recipients, patients with hematological malignancies and other chronically immunosuppressed populations, hCMV reactivation can lead to severe complications, including pneumonia, hepatitis, gastrointestinal disease, graft rejection, and increased mortality.^3^ Moreover, hCMV can seriously impact fetal development, notoriously leading to sensorineural hearing deficits.^4^^,^^5^ These risks underscore the need for innovative diagnostic approaches that ultimately support timely and accurate hCMV detection.

Low-complexity PoC testing, potentially even in the form of at-home or self-monitoring tools could reduce the need for frequent hospital visits. Current diagnostic methods include serology, which has limited sensitivity (and limited applicability in case of non-primary infection), and quantitative PCR (qPCR), often supplemented with sequencing for antiviral resistance screening. qPCR and sequencing typically target conserved hCMV genes *UL54* and *UL55*.^6^^,^^7^ While effective, these methods require certified laboratories, *in vitro* diagnostics regulation (IVDR)-approved platforms, and evolving commercial kits, making testing costly, labor-intensive, and increasing turnaround times.

Clinically relevant hCMV levels in patient plasma vary broadly, down to attomolar levels, corresponding to roughly 10^3^–10^5^ copies/mL.^8^ Isothermal amplification techniques such as recombinase polymerase amplification (RPA)^9^ and loop-mediated isothermal amplification (LAMP)^10^ have been explored as alternatives to PCR, reducing reliance on centralized laboratories and specialized equipment, enabling field-deployable diagnostics. However, these methods have their drawbacks for the detection of hCMV DNA in plasma, as it is highly fragmented.^11^ Due to the need for long (around 35 nt) or multiple (>2) primers, a significant portion of hCMV DNA fragments in plasma will not be amplified as they are <100 bp.^12^ Moreover, RPA and LAMP challenge accurate target quantification, as amplification is not cycled.

Given these limitations, we here explore a proof-of-concept amplification-free CRISPR-Cas12a-based approach for hCMV detection. Clustered regularly interspaced short palindromic repeats (CRISPR) and CRISPR-associated (Cas) proteins form RNA-guided adaptive antiviral defense systems in bacteria. Recent characterization of type V (Cas12) and type VI (Cas13) systems revealed their indiscriminate RNA-guided *trans*-cleavage activities, enabling isothermal nucleic acid detection at a cost-effective rate.^13^ Furthermore, with spacers of ∼20 nt, CRISPR-based diagnostics (CRISPRdx) can efficiently target fragmented DNA.^14^ As a starting point for considering suitable PoC formats, we design and implement a CRISPRdx hCMV assay on a lateral flow assay (LFA) with a simple chromogenic readout for quick interpretation of test results. However, as the typical amplification-free picomolar detection limit^15^ of CRISPRdx is insufficient for early hCMV detection, we initially use it in conjunction with PCR to allow testing on clinical samples.

Moving toward amplification-free testing, sensitivity was first improved by multiplexing up to 10 targets on different viral loci. The inherent signal amplification of Cas12a *trans*-cleavage was then improved through optimizing several reaction conditions, including reporter sequence. Finally, we explore amplification-free quantitative sensing at clinical concentrations in an experimental PoC format, translating the achieved sensitive assay into a microfluidic digital droplet chip as a strategy for quantification by isolating target molecules into picoliter droplets.

## Results

### Dual-multiplexed detection of hCMV

**Figure 1:**
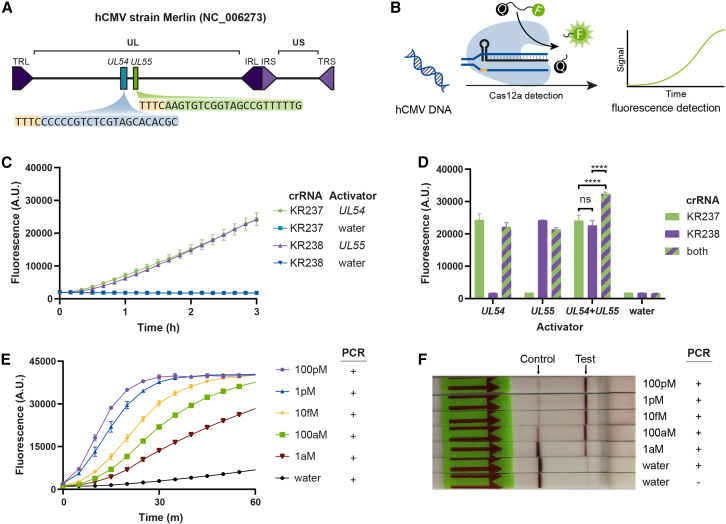
Cas12a dual multiplexed detection of hCMV (A) hCMV strain Merlin genomic sequence (GenBank accession no. NC_006273) was used to design crRNAs targeting *UL54* and *UL55*. Target site sequences and preceding protospacer adjacent motifs are shown 5′→3′. (B) Cas12a collateral activity can be harnessed for specific target-dependent fluorophore release to visualize hCMV-positive samples. (C) Singleplexed CRISPR-based detection of 100 pM synthetic *UL54* and *UL55* activators. (D) Endpoint fluorescence values after 3 h multiplexed detection of 100 pM synthetic *UL54* and *UL55* activators. (E) Multiplexed detection of PCR-amplified *UL54* and *UL55* can detect down to 1 aM within 60 min. (F) Multiplexed Cas12a-based LFA detection of *UL54* and *UL55*. Positive samples produce increased test band visibility and reduced control band intensity. All graphs display error bars indicating standard error of the mean of triplicate reactions. Relevant significant differences (*p* ≤ 0.0001) are indicated with ∗∗∗∗, as calculated through two-way ANOVA.

To detect hCMV in human blood, we designed crRNAs that target the viral genome at unique, hCMV-specific sequences. hCMV harbors a 236 kb linear dsDNA genome, composed of a unique long (UL) and a unique short (US) region, enclosed by terminal/internal repeats flanking the long/short region (TRL/IRL/TRS/IRS) (Figure 1A). crRNAs KR237 and KR238 were designed for Cas12a-based detection in conserved genes *UL54* and *UL55*, respectively, which are commonly targeted by qPCR assays. In the presence of target DNA, Cas12a-crRNA complexes induce collateral cleavage of a fluorophore-quenched ssDNA reporter, generating a fluorescence signal (Figure 1B).

Comparing LbCas12a and AsCas12a Ultra under non-optimized reaction conditions, we found that AsCas12a Ultra exhibited superior *trans*-cleavage kinetics,^16^ producing an increased fluorescence signal with shorter reaction times (Figure S1C). Without amplification, AsCas12a Ultra detected 100 pM hCMV DNA within an hour (Figure 1C) and 10 pM detection required at least 3 h (Figure S1C). Sensing 10 pM with LbCas12a produced a signal less than half as strong as that of AsCas12a, so that detection is difficult to distinguish from the baseline, even after 5 h (Figure S1C). Multiplexed amplification-free detection targeting both *UL54* and *UL55* significantly accelerated fluorescence kinetics when detecting a 1:1 mixture of both targets (Figure 1D).

However, the 10 pM sensitivity remains insufficient for direct hCMV detection in clinical samples with concentrations in the femtomolar range and below.^11^ To bridge this sensitivity gap, we incorporated a multiplexed PCR pre-amplification step, enabling 1 aM plasmid DNA detection within an hour (Figure 1E). This clinically relevant sensitivity was validated using qPCR on clinical isolates (Figure S2). To illustrate how Cas12a can be used in a PoC format, a readout into a paper-based LFA for colorimetric detection was used (Figure 1F).

### Further increasing sensitivity by higher-level multiplexing

**Figure 2:**
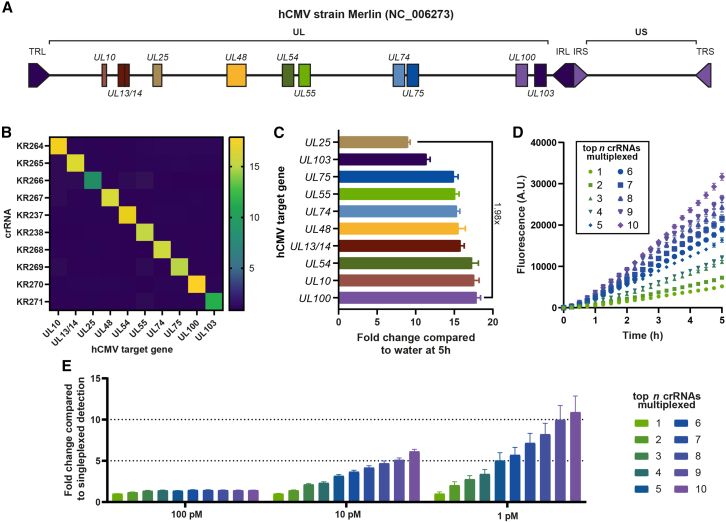
Amplification-free multiplexed detection of hCMV with up to 10 crRNAs significantly improves signal-to-noise ratio compared to singleplexed detection (A) Schematic overview of 10 different target loci in the unique long region of the hCMV genome. One target selected target site detects the junction of *UL13* and *UL14*. (B) Heatmap displaying the mean signal-to-noise ratio of a triplicate cross-activity assay, using 1 nM target for 5 h. (C) Signal-to-noise ratio for each pair of crRNA and intended target, using 1 nM target for 5 h. (D) Multiplexed detection of 10 pM in-reaction concentration hCMV. (E) Signal-to-noise ratio for multiplexed detection after 5 h of incubation when using 100, 10, or 1 pM of in-reaction activator cocktails. Error bars indicate standard error of the mean of triplicate reactions. crRNA cocktails were kept at 25 nM total RNA in-reaction concentration. All used activator cocktails consist of equimolar amounts of all 10 targets, and the reported concentrations represent the level of each target.

The remainder of this work explores sensitivity-optimization under amplification-free conditions. Based on the observed sensitivity improvement when multiplexing two crRNAs (Figure 1D), we hypothesized that adding more crRNAs could increase sensitivity even further, reducing the need for target pre-amplification. To test this, we designed an additional eight crRNAs, bringing the total to 10 targets: *UL10*, *UL13/14*, *UL25*, *UL48*, *UL54*, *UL55*, *UL74*, *UL75*, *UL100*, and *UL103—*all known conserved open reading frames (ORFs) among characterized hCMV strains (Figure 2A).

To confirm specificity, all 100 possible crRNA-activator combinations were tested without amplification (Figure 2B), revealing variation in signal generation across targets. For instance, *UL100* yielded nearly twice the signal-to-noise ratio of *UL25* (Figure 2C). We ranked crRNAs by activity in singleplex and formulated crRNA cocktails with increasing degrees of multiplexing, beginning with the most active crRNA (e.g., cocktail 1 targets *UL100* only; cocktail 2 targets *UL100* and *UL10*; cocktail 3 targets *UL100*, *UL10*, and *UL54* etc.). Each reaction received the same activator cocktail containing equimolar amounts of all 10 targets to mimic fragmented hCMV DNA. Amplification-free multiplexing significantly improved signal strength compared to singleplex detection, particularly at lower activator concentrations (Figures 2D and 2E). These findings demonstrate that crRNA cocktails are a viable strategy to enhance test sensitivity, making them a promising approach for detecting low-abundance target DNA. Although individual crRNA performances are unequal, we observed a near-linear increase in endpoint fluorescence fold change compared to singleplexed detection when testing for target levels under 100 pM (Figure 2E). Compared to a reaction with *n*-1 crRNAs, the percentual endpoint fluorescence increase in a reaction with *n* crRNAs seems to decrease less fast at lower target levels (Figure S3), suggesting that higher order multiplexing particularly benefits detection of ultra-low level targets. In these low-concentration conditions, we observed a >10-fold change for decamultiplexed detection of 10 pM target, compared to ±6-fold change for decamultiplexed detection of 100 pM target (Figure 2E).

### Optimizing the reporter sequence and reaction conditions allows amplification-free femtomolar detection

**Figure 3:**
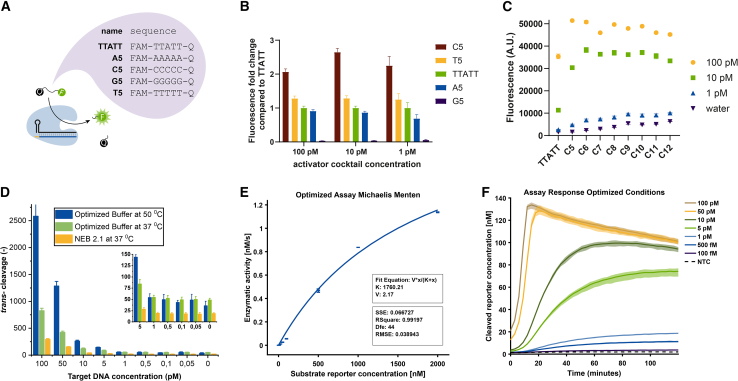
Cytosine-rich probes result in faster fluorescence growth rates (A) Schematic overview of the tested quenched fluorescent probes. All probes have a 5′ FAM group, a 3′ quencher [Q] group, and only differ in DNA sequence. (B) Fluorescence endpoint fold change at 1 h incubation, compared to using TTATT probe, based on three technical replicates, error bars display standard error of the mean. (C) Effect of varying the probe length on the fluorescence background. Based on raw endpoint fluorescence values from three technical replicates after 3 h of incubation, error bars display standard error of the mean. Data are not background-corrected. (D) Comparison of the assay’s *trans*-cleavage activity between the optimized and previous reaction conditions. Data are represented as mean ± standard error of the difference between the means of three absolute and three technical replicates (*n* = 9). (E) Fit of the Michaelis-Menten equation for the assay with optimized condition: the reactions were performed at 50°C using the optimized buffer and 6C reporter. Error bars show the mean and standard deviation of two absolute and three technical replicates (*n* = 6). (F) Response of the assay to different in-reaction target concentrations, carried out at optimized reaction conditions. Error bars show the minimum and maximum readout of three technical replicates.

We optimized reporter design, buffer composition, and reaction temperature to accelerate detection further and improve assay sensitivity. The conventional 5′TTATT3′ ssDNA quenched FAM probe widely used in Cas12a-based diagnostics,^15^ was compared to four different homopentanucleotide probes (A5, C5, G5, and T5) (Figure 3A) in an amplification-free 10-crRNA multiplexed setup with 10 pM target DNA. Among these, the C5 probe performed best, reaching the fluorescence plateau within 5 h (Figure S4). At lower target concentrations, C5 exhibited a 2-fold increase in fluorescence within the first hour compared to TTATT (Figure 3B), suggesting a bias in nucleotide preference.

Besides its nucleotide preference, we reasoned that the indiscriminate Cas12a collateral activity may prefer to cleave reporters with more accessible phosphorothioate bonds. Increased probe length could offer the Cas12a RuvC active site multiple options to cleave a probe. Testing longer polycytosine reporters (6–12 nt) revealed that these probes indeed further improved fluorescence intensity and signal growth rate nearly up to 5-fold compared to the TTATT probe, but at the cost of elevated background noise (Figure 3C; Figure S5). A C6 probe provided the best balance between sensitivity and specificity (Figure 3C), achieving a higher endpoint fluorescence than the shorter C5 probe which may be attributed to a higher accessibility for cleavage. Additional reporter designs reported in literature, including hairpin structures^17^ and TTATT-5C extensions,^18^ were tested but exhibited significant background activity at low target concentrations (Figure S6).

With the 6C reporter, we systematically optimized the reaction buffer and temperature for AsCas12a Ultra. Using a 384-well plate assay with a constant 1 nM target, we measured *trans*-cleavage kinetics by recording fluorescence over time for different buffer compositions (varying Na^+^, Mg^2+^, DTT, Tris-HCl, and PEG; see Table S3) and temperatures (Figures S6 and S7). Our optimized buffer contains 15 mM Mg^2+^, 10 mM Tris-HCl, no Na^+^, 2 mM DTT, 2.5% PEG, buffered at pH 8.5. At 50°C, this improved the initial fluorescence increase by 3× compared to NEB2.1 buffer at 37°C, with an additional 3× gain observed at 50°C, yielding an overall 10× improvement (Figure 3D). We quantified the enzyme performance of this optimized reaction, combining the enhancement from the 6C reporter, adjusted buffer composition and elevated 50°C temperature. We first characterized the Michaelis-Menten kinetics of the reaction (Figure 3E), finding the Michaelis constant K_m_ = 1760 nM and a limiting rate of V_max_ = 2.16 s^−1^, indicating that under the optimized conditions Cas12a attains near optimal cleavage performance under conditions where substrate concentrations are in the low micromolar range. In addition, bulk assays across a range of target concentrations estimated a *trans*-cleavage signal amplification factor in the order of 10^4^, with 20 nM Cas12a converting femtomolar target concentrations into a detectable nanomolar fluorescence signal (Figure 3F).

### Application of the optimized assay in a digital droplet chip

In addition to the LFA, which employs PCR amplification, we explored the use of isothermal, amplification-free methods for simplified, quantitative sensing of hCMV at clinical concentrations. The bulk reaction, as optimized in previous sections, was applied to perform a digital droplet assay without target amplification through partitioning into miniature volumes. Within droplets that contain a target, the fluorescent output of reactions is confined to a chosen droplet volume, so that the reaction takes place at an increased local concentration. This in-droplet concentration is independent of the sample volume, which can instead be estimated from the fraction of positive droplets.

**Figure 4:**
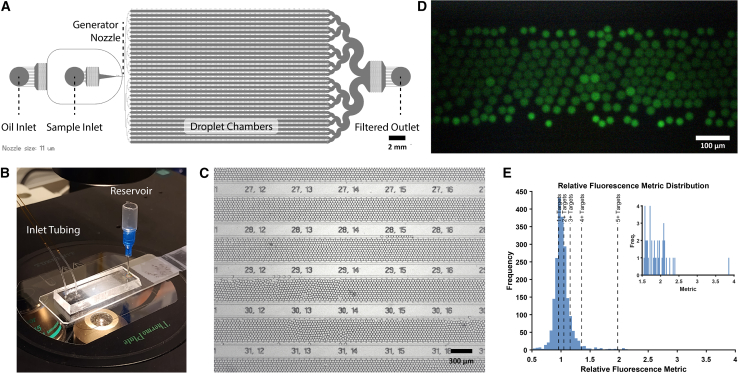
Application of the optimized assay in a digital droplet chip (A) Top view of the chip layout consisting of a flow-focusing droplet generator and a series of chambers to store and observe the droplets during the assay. The whole chip was first filled from the outlet on the right, then droplets are generated to fill the droplet chambers from the left. (B) Photograph of the chip in the imaging setup. (C) Microscopy image of generated monodisperse droplets in the chambers before heating. The scale bar indicates 300 μm. (D) Fluorescence image of a channel with droplets after heating. The scale bar indicates 100 μm. (E) Histogram showing the droplet mean endpoint fluorescence distribution relative to their neighbors using droplets from within a single channel (*n* = 2,072). Vertical lines show the theoretical fractions that, based on the known sample concentration, should have contained at least a certain number of targets. The inset shows a zoom of the droplets with a metric greater than 1.5.

We designed and fabricated a microfluidic chip containing a flow-focusing droplet generator to generate water in oil (w/o) droplets, which are then distributed over a series of parallel running chambers for incubation and imaging (Figures 4A and 4B). Stable, monodisperse droplets (diameter 23.00 ± 0.52 μm, based on 7.8 × 10^3^ droplets; Figure S12) formed within minutes by adjusting inlet pressures (Figure 4C). An oil reservoir was connected to prevent evaporation. Instead of 50°C, droplets were incubated at a limited 44°C as a compromise considering practical constraints regarding the droplet stability in the setup. The chip was subsequently imaged under fluorescent light after 2 h of incubation (Figure 4D). While droplets remained compartmentalized, droplets decreased in radius over this time, especially near the channel edges (diameter 17.10 ±1.84 μm, based on *n =* 7.3 × 10^3^; Figure S12A). Since droplet shrinkage depended on chip location, it was likely caused by hydrostatic pressure imbalances and thermally induced passive flows.

A sample reaction mixture containing 4 pM of target was chosen to achieve a balance of positive and negative droplets. Analysis on a subset of stable, monodisperse droplets (diameter 21.4 ±1.3 μm, *n* = 2072) revealed that the distribution of droplet fluorescence followed a skewed distribution rather than a bimodal distribution. As this indicates that the kinetics in droplets is insufficient, we use Poisson statistics to estimate the remaining gap. For this, a thresholding metric identified droplets exhibiting fluorescence ≥1.5× above the background (Figure 4E), a threshold where positive identification could clearly be validated by visual inspection of the results. With this metric, only 1.98% (41 out of 2072 droplets) is above the threshold. Comparing this percentage to distribution predicted by Poisson statistics based on the known input concentration and initial droplet size, we find that 4.3% of all droplets should contain at least four targets. Thus, while signals were detectable, they primarily originate from droplets containing >4–5 target molecules, corresponding to a 1.24–1.55 pM local concentration. This indicates that the sensing and imaging method works without amplification, but also that, beyond the kinetic improvements, additional measures may be needed for the single molecule detection required for digital droplet assays capable of measuring ultra-low viral loads.

## Discussion

Detecting hCMV in clinical samples is challenging due to the ultra-low concentrations and DNA fragmentation. Conventional Cas12a-based sensing is limited by enzyme kinetics and signal dilution. We improved the sensing capabilities of AsCas12a Ultra-assisted assays using standard components and without amplification. At low target concentrations, multiplexing up to 10 crRNAs resulted in near-linear increases in signal intensity. Optimizing the bulk assay without multiplexing, we can detect femtomolar concentrations within an hour. We applied a dual-guide multiplexed reaction in a lateral flow assay to sense attomolar concentrations employing PCR. Furthermore, the optimized reaction in a proof-of-concept digital droplet chip converts <1.84 pM local concentration of target into a detectable signal, which, while insufficient for quantification, suggests that amplification-free detection of attomolar concentrations in PoC formats is within reach.

Multiplexing resulted in a near-linear sensitivity increase at low concentrations, demonstrated in a dual-multiplexed lateral-flow assay on clinical isolates. The as much as 10-fold signal improvement contrasts with findings by Chen et al., where multiplexing beyond four guides reduced efficiency.^19^ The observation that the 5^th^ and 6^th^ crRNA from Chen et al. had poor on-target activity suggests that unfit crRNAs occupy Cas12a proteins and hamper the synergistic effects, so that multiplexing only benefits the overall kinetics if the pool is made up of fit crRNAs. Our use of computationally optimized crRNAs^20^ likely prevented this bottleneck. Notably, the per guide-signal improvement in multiplex does not reflect the per-guide activity in singleplex, which could be due to unequal crRNA adoption by the Cas12a enzyme. We also found that multiplexing was most efficient at lower target levels. At a higher concentration, singleplexing is already nearly sufficient to reach saturation quickly. At lower concentrations, only subsets of the viral genome may be present. Multiplexing therefore increases the likelihood of target recognition and cumulatively contributes to signal yield. The near-linear signal improvement with multiplexing offers a promising strategy to lower the LoD in rare-event sensing. This aligns with the findings from Zeng and colleagues, who demonstrated enhanced amplification-free detection of African swine fever virus.^21^ Beyond fragmented target detection, multiplexing may also aid in targeting highly mutated or strain-dependent sites by reducing the risk of decreased binding because of mismatches.

In optimizing the reaction components, we selected AsCas12a Ultra, a dual-mutant variant originally derived through evolutionary engineering for enhanced genome editing efficiency.^22^ This increased *cis*-activity likely leads to faster ribonucleoprotein activation, thereby resulting in its superior kinetics in *trans*. Most studies evaluating alternatives to the standard TTATT probe employ LbCas12a.^18^^,^^19^^,^^23^ AsCas12a preferentially cleaved cytosine-rich probes over thymine- or adenine-rich probes, which corresponds to recent findings by others.^23^ Guanine-rich probes showed minimal fluorescence, which might be explained by strong quenching of FAM dyes by guanine overhangs.^24^^,^^25^^,^^26^ In a less pronounced effect, the cytosine nucleotides also appear capable of quenching, but this was only for longer reporter sequences (Figure S5B). Additionally, while longer probes are cleaved faster, the increased distance between the fluorophore-quencher pair decreases the quenching efficiency. We optimized this trade-off using a 6-nt cytosine homopolymer reporter, boosting the rate of fluorescence increase 4-fold compared to the standard TTATT probe.

When optimizing reaction conditions, we found AsCas12a Ultra to be exceptionally stable at elevated temperatures with an optimal temperature of 50°C, accelerating reaction rates. The resulting kinetic constants K_m_ = 1.76 μM and V_max_ = 2.16 s^−1^ we found are favorable for amplification-free sensing, allowing us to detect femtomolar hCMV material in bulk. This substantially improves upon the parameters V_max_ = 0.6 s^−1^ and K_m_ = 2.7 μM found by Ramachandran et al. for wild-type AsCas12a under unoptimized conditions.^27^ In the same work, the authors report that parameters in literature often appear unrealistically high. We therefore validate our kinetics using their presented back-of-the-envelope (Tables S4 and S5). Notably, the achieved kinetics is comparable to that of LbCas12a orthologs optimized for *trans*-cleavage as reported by others.^28^^,^^29^ We demonstrate a high femtomolar LoD in bulk where AsCas12a-assisted assays typically reach low picomolar concentrations without additional techniques to improve detection.^30^

There is a merit to quantitative sensing of lower concentrations, even reaching an attomolar LoD. Such concentrations are relevant, for instance, in prenatal hCMV monitoring. hCMV can impact fetal development, often leading to deafness, neurosensory impairments,^4^^,^^5^ learning deficits, and even psychiatric disorders.^31^ Early gestational infections may result in fetal growth restriction and even spontaneous abortion.^32^ These severe implications prioritize hCMV as a critical target for accurate and timely diagnosis. However, it is often unclear when a hCMV infection poses a clinical threat and needs to be treated. For instance, while treatment options during pregnancy are available,^33^^,^^34^^,^^35^ and early detection is crucial for effective treatment,^36^ the viral load threshold for treatment remains undetermined.^37^^,^^38^ The recent finding that hCMV infections can be detected during sequencing-based noninvasive prenatal testing (NIPT) for fetal aneuploidies demonstrates that hCMV could be monitored starting from early gestation.^12^^,^^39^ Follow-up monitoring the viral load over time, or screening to determine a clinical threshold for treatment, would only be possible if sufficiently low-cost, point-of-care (PoC) diagnostic tools are devised with accuracy, ultra-low sensitivity, and quantitative results to be commutable with qPCR.

Our proof-of-concept test applying the optimized assay in a digital droplet chip could prove a step forward toward this goal. While we did not achieve single-molecule amplification, earlier work by Yue et al. reports amplification-free attomolar sensitivity^40^ using a similar digital droplet chip and clinical samples, although using LbCas12a and targeting a different application where dual-guide strategies unsuitable for fragmented DNA could be used. Our results indicating the proximity to digital readout without multiplexing or amplification highlight the potential of amplification-free digital assays in comparison to bulk formats. Both digital assays and amplified bulk CRISPRdx assays can achieve attomolar LoDs competing with qPCR,^14^ but the quantitative nature of digital assays sets them apart from the bulk reaction.

Based on our findings, a combination of smaller partitions and multiplexing may allow quantification of attomolar concentrations similar to qPCR for hCMV in the future (Figures S13 and S14). Multiplexing would increase the number of positive droplets, reducing the required assay volume. Smaller partitions could then enable single-molecule detection with the kinetics achieved in this study. This would require the development of a device capable of heating small partitions for an extended time without droplet volume loss.

In conclusion, using guide multiplexing under optimized conditions achieves a sensitivity increase desirable for CRISPR-Cas12a-based diagnostics. The achieved femtomolar detection limit overlaps with high viral-load cases. Further improvements in reaction kinetics may enable quantitative amplification-free PoC sensing of ultra-low viral loads. These findings support the development of quantitative CRISPR-based diagnostics for monitoring low-abundance viral DNA in clinical settings.

### Limitations of the study

While we were able to show the effectiveness of using up to 10 guides, the question remains as to whether there is a point beyond which multiplexing becomes ineffective. Aside from physical limitations, each additional guide causes a proportionally smaller signal improvement relative to the singleplex assay (Figure S3), while the work required to design and test the crRNA pool increases with each crRNA. Despite the tools available to facilitate designing fit guides, there are therefore practical limitations to the amount of guides that can reasonably be used.

While initial tests of the Cas12a-assisted test could be performed on clinical isolates, the proposed digital assay could not yet be applied to biological samples. Although the use of synthetic targets and isolates provides valuable insight into the kinetics of the assay, performance is typically reduced in clinical samples due to effects of active enzymes or competing genetic material that may be found in samples. Eventual clinical validations will have to consider sample types (e.g., blood, urine, saliva, etc.), required pre-processing steps, and corresponding expected target ranges to determine a clinically relevant LoD. Under such conditions, synthetic targets may still function as positive or internal controls.

While the optimization of our assay achieves measurable improvement in bulk, it is possible that there is a difference in optimal conditions in droplets compared to bulk. There may be physical differences (for instance related to transport) or technological limitations. One prominent example of such limitations is the assay temperature. During the course of this research, we noticed challenges in droplet stability in the chips fabricated out of rapid-prototyping materials. This stability was influenced by the temperature of the device, accelerating the shrinking of droplets. Such limitations would have to be overcome or taken into account when translating the assay to a format suitable for clinical testing.

We further acknowledge the remaining work required in translating the device to a commercially applicable device. Such work may include changes to material choices and fabrication methods, testing of room temperature stability of lyophilized reagents, or development of readout equipment. While droplet-based assays are often used in experimental work because of their compatibility with rapid-prototyping workflows, their complexity limits their immediate use in PoC settings. However, the conceptual results are transferable to other digital assay types, such as assays using partitions in chambers or nanowells, which can be more suitable for PoC settings.

Because our digital assay was limited by the achieved assay kinetics, we did not apply multiplexing to the digital assay. While multiplexing will not improve the kinetics within a single droplet, considering that the different fragments targeted by different crRNAs are distributed over different droplets, multiplexing theoretically allows achieving a positive test in a greater number of droplets. In Figure S13 we show, using a statistical model, how this benefits the statistics of the assay such that an LoD in the order of 10^−17^ M can theoretically be reached with the kinetics achieved in this paper. One limitation of this approach is that the large number of empty partitions increases the sensitivity to false positives. Without further kinetics optimizations, future amplification-free digital assays for PoC may search for low-complexity methods to increase signal strength even in larger droplets with lower local target levels.

## Resource availability

### Lead contact

Requests for further information and resources should be directed to and will be fulfilled by the lead contact, Kavish A.V. Kohabir (k.a.v.kohabir@amsterdamumc.nl).

### Materials availability

Plasmids generated in this study are available from the lead contact with a completed materials transfer agreement.

### Data and code availability

• Data reported in this paper will be shared by the lead contact upon request.
• All original MATLAB code has been deposited at GitHub at https://github.com/JasperR-UT/Codebase-AUMC-UT-Multiplex-Digital-Cas12a/tree/master and is publicly available as of the date of publication.
• Any additional information required to reanalyze the data reported in this paper is available from the lead contact upon request.

## Acknowledgments

A.W.J.R., J.E.v.D., and L.I.S. acknowledge support from the Dutch National Growth Fund program NXTGEN Hightech.

## Author contributions

Conceptualization, K.A.V.K., A.W.J.R., J.E.v.D., and J.L.; methodology, K.A.V.K., A.W.J.R., and J.E.v.D.; investigation, K.A.V.K., A.W.J.R., L.O.N., R.E.B., and J.E.v.D; writing – original draft, K.A.V.K. and A.W.J.R; writing – review and editing, J.E.v.D., J.L., R.M.F.W., M.R.A.W., L.I.S., and E.A.S.; funding acquisition, R.M.F.W., L.I.S., and E.A.S; resources, M.J. and M.R.A.W.; supervision, J.E.v.D., J.L., R.M.F.W., L.I.S., and E.A.S.

## Declaration of interests

J.E.v.D. and L.I.S. are inventors on two patents related to CRISPR/Cas diagnostic technology (P35877NL00 and P35878NL00).

## Declaration of generative AI and AI-assisted technologies in the writing process

During the preparation of this work, the authors used Grammarly in order to improve grammar and make the sentences more readable. After using this tool/service, the authors reviewed and edited the content as needed and take full responsibility for the content of the publication.

## STAR★Methods

### Key resources table

**Table:** 

REAGENT or RESOURCE	SOURCE	IDENTIFIER
**Bacterial and virus strains**		

*Escherichia coli* subcloning efficiency DH5α competent cells	Invitrogen™	18265017

**Chemicals, peptides, and recombinant proteins**		

Difco™ LB broth, Lennox	Becton, Dickinson & co.	SKU 240230
Difco™ Agar Noble	Becton, Dickinson & co.	SKU 214230
Kanamycin	Sigma-Aldrich	K1377-25G
Q5® Hot Start High-Fidelity DNA Polymerase	New England Biolabs	M0493L
Polydimethylsiloxane (PDMS, SYLGARD™ 184 Silicone Elastomer)	Dow Inc.	4019862
Aquapel®	PPG Industries Inc.	N/A
rCutSmart® buffer	New England Biolabs	B6004S
NEBuffer 2.1	New England Biolabs	B6002S
AsCas12a Ultra	Integrated DNA Technologies Inc.	10001273
FluoSurf	Emulseo	P/*N* 3200808

**Critical commercial assays**		

Qubit™ dsDNA HS kit	Thermo Fisher Scientific	Q32851
Zero Blunt™ PCR Cloning Kit	Thermo Fisher Scientific	K270020
Mix2Seq kit	Eurofins Scientific	3094-000MSK
Pure Yield™ Plasmid Midiprep system	Promega Corp.	A2495
GenLine HybriDetect	Milenia Biotech	MGHD 1

**Deposited data**		

Original MATLAB code	This paper	https://github.com/JasperR-UT/Codebase-AUMC-UT-Multiplex-Digital-Cas12a/tree/master
hCMV Merlin strain reference genome	NCBI	GenBank Accession no. NC_006273

**Oligonucleotides**		

Oligonucleotides	This paper	See Table S1

**Recombinant DNA**		

Plasmid pZeroBlunt-CMV_UL54+UL55	This paper	N/A

**Software and algorithms**		

CHOPCHOP v3	Labun et al. 2019	RRID: SCR_015723
SnapGene v8	Dotmatics	RRID: SCR_015052
Graphpad Prism	Dotmatics	RRID: SCR_002798
Standard nucleotide BLAST (megablast)	NCBI	RRID: SCR_004870
CleWin	WieWeb software	N/A
MATLAB (R2023b)	The MathWorks	RRID: SCR_001622
i-control™	Tecan Group Ltd.	RRID: SCR_024562
Microsoft Excel	Microsoft Corp.	RRID: SCR_016137
OriginPro 2023	OriginLab Corp.	RRID: SCR: 015636

**Other**		

Infinite® 200 Pro M Plex plate reader	Tecan Group Ltd.	RRID: SCR_020543
MagNA pure 96	Roche Diagnostics	N/A
Qubit™ 4 Fluorometer	Thermo Fischer Scientific	Q33238; RRID: SCR_026883
Heraeus stationary incubator	Thermo Fischer Scientific	RRID: SCR_024558
SpectraMax ID3 microplate reader	Molecular Devices	RRID: SCR_023920
CUTE plasma processing system	Femto Science	N/A
LineUp™ pressure-driven pump	Fluigent	LU-FEZ-1000, LU-LNK-0001; RRID: SCR_021145

### Experimental model and study participant details

hCMV-positive clinical left-over nucleic acid extraction eluates from EDTA-whole blood samples were obtained as part of routine clinical diagnostic testing for hCMV at the Amsterdam UMC, Department of Medical Microbiology and Infection Prevention. For all patient samples, patient records were verified for consent to re-use left-over clinical materials. Samples from individuals who had not opted out were fully anonymized prior to reuse in this study, in accordance with national and institutional guidelines. Ethical clearance was obtained from the Amsterdam UMC Medical Research Ethics Committee (Reference no.: 2025.0014).

### Method details

#### Target selection

hCMV DNA target sequences were identified using the hCMV Merlin strain (GenBank accession no. NC_006273) as input sequence in CHOPCHOP v3.^20^ The human reference genome (GRCh38) was used to identify off-target loci with up to three mismatches. Among the candidate hits, the top efficiency scoring crRNAs were selected, and the spacers were analyzed with standard nucleotide BLAST,^41^ excluding “Human cytomegalovirus CMV” (taxid:10359) to ensure hCMV-specific spacers that do not overlap with other genomes. The resulting set of on-targets included *UL10, UL13/14, UL25, UL48, UL54, UL55, UL74, UL75, UL100* and *UL103*, which are all conserved open reading frames (ORFs) among characterized hCMV strains.^42^

#### Oligonucleotide preparation

Synthetic DNA and RNA oligonucleotides were ordered from Integrated DNA Technologies, Inc. (IDT, Coralville, USA) and Eurofins Genomics (Ebersberg, Germany). Their sequences are listed in Table S1. Ordered AsCas12a crRNAs were reconstituted in nuclease-free water to 100 μM. crRNA cocktails were prepared through equimolar mixing of crRNAs, keeping the total level of crRNA constant for each cocktail unless otherwise stated. crRNA solutions were stored at –20 °C until further use. Short 55 bp dsDNA activators were formed by annealing reverse complementary synthetic ssDNA oligonucleotides in equimolar amounts. Briefly, the nucleic acid mixtures were mixed into a 1x buffer, heated to 95 °C for 2 min, and cooled down 0.1 °C/s to ambient temperature for duplex DNA formation. The annealed product was quantified using the Qubit™ dsDNA HS kit (Invitrogen™, Thermo Fisher Scientific) on a Qubit Fluorometer (Invitrogen™, Thermo Fisher Scientific). Additional buffered dilutions were prepared and stored at –20 °C until further use. Activator cocktails were prepared by equimolar mixing, taking into account that this dilutes each target within the total cocktail volume (e.g. an activator cocktail of 1 nM has 1 nM of each component, effectively mimicking a 1 nM fragmented hCMV DNA isolate). In case of patient samples, genomic DNA was extracted from 24 clinical EDTA-whole blood samples using a MagNA pure 96 instrument (Roche Diagnostics GmbH, Mannheim, Germany), according to the manufacturer’s instructions, with a total elution volume of 50 μL for 200 μL samples.

#### Plasmid cloning & isolation

Cloning and propagation of plasmids harboring *UL54* and *UL55* target sites was done in Escherichia coli subcloning efficiency DH5α competent cells (Invitrogen™, Thermo Fisher Scientific). According to the manufacturer’s instructions, a 500 bp dsDNA oligo (KD181) consisting of 250 bp of each hCMV gene was inserted into the provided backbone of the Zero Blunt™ PCR Cloning Kit (Thermo Fisher Scientific). Heat-shock transformation was performed for 45 s at 42 °C, followed by immediate cooling on ice, one-hour recovery in non-selective medium, plating on solid Luria-Bertani (LB) medium, supplemented with 50 μg/mL kanamycin, and overnight growth at 37 °C in a Heraeus stationary incubator (Thermo Fisher Scientific). Constructs were verified by gel electrophoresis size analysis of colony PCR products and by Sanger sequencing using the Mix2Seq kit (Eurofins Scientific). For plasmid isolation, a 200 mL selective liquid culture was inoculated from a verified single bacterial clone and grown overnight at 37 °C, at 200 rpm in a Unitron® plus incubator shaker (Infors HT, Switzerland). Plasmids were harvested using the Pure Yield™ Plasmid Midiprep system (Promega Corporation, Madison, USA) and quantified using a NanoDrop™ One microvolume Spectrophotometer (Thermo Fisher Scientific). Dilutes were quantified using the Qubit™ dsDNA HS kit (Invitrogen™, Thermo Fisher Scientific) on a Qubit™ 4 Fluorometer (Invitrogen™, Thermo Fisher Scientific).

#### Nucleic acid amplification

Dual multiplexed PCR on *UL54* and *UL55* was done using primers KD183, KD187, KD193 and KD196 with Q5® Hot Start High-Fidelity DNA Polymerase (New England Biolabs), keeping the total concentration of primers according to the manufacturer’s instructions. Target plasmid cloning & isolation is described above. Thermocycling was done for 30 cycles, with an annealing temperature of 68 °C. Reaction mixtures were directly used as activator in collateral cleavage assays.

#### Fluorescent reporter assays

Bulk detection *trans-*cleavage reactions were performed as described previously.^43^^,^^44^ Reactions contained 1× rCutSmart® buffer, 1x NEBuffer 2.1 (New England Biolabs), or optimized buffer, depending on the experiment. Unless otherwise denoted, experiments used 1 μM ssDNA probe labeled with an Iowa Black®-quenched 6-carboxyfluorescein (6-FAM) dye, 20 nM AsCas12a Ultra (Integrated DNA Technologies, Inc. IDT, Coralville, USA) and 25 nM crRNA. Oligonucleotide preparation and nucleic acid amplification are described above. Reactions were initiated by adding short synthetic dsDNA activators to 20 μL final volume or by adding 2 μL of a raw PCR reaction mixture. In the case of ribonucleoprotein (RNP) pre-formation, Cas12a and crRNA were mixed in a 1:1.25 ratio in buffer and incubated at 37°C for 30 minutes to form a 9 μM RNP solution. The RNP solution was subsequently diluted for use in preparing *trans-*cleavage reactions. Reactions were incubated at 37°C for an indicated amount of time while monitoring fluorescence (λ_ex_ = 485 nm, λ_em_ = 535 nm) periodically in an Infinite® 200 Pro M Plex plate reader (Tecan Group Ltd., Männedorf, Switzerland) or SpectraMax ID3 microplate reader (Molecular Devices, San Jose, CA, USA). Plate reader settings are listed in Table S2. Unless stated otherwise, target concentrations represent final in-reaction levels.

#### Lateral flow assays (LFAs)

*Trans*-cleavage reactions were upscaled to 50 μL end volumes in 0.2 mL PCR tubes for LFAs. Instead of 1 μM quenched fluorescent probe (KD035), reactions were complemented with 100 nM biotin-labeled probe (KD220). After adding the activator, reactions were incubated for 30 minutes at 37 °C prior to the insertion of conjugate release pads of Milenia® GenLine HybriDetect universal LFA strips (Milenia Biotech, Gießen, Germany). Strips were imaged after 5 minutes of lateral flow.

#### Reaction condition optimization

*Trans*-cleavage activity was optimized by changing one variable at a time (Table S3; Figure S7). The 1 nM target concentration, 20 nM RNP complex concentration, and a reporter concentration of 1000 nM were kept constant. *Trans*-cleavage rates were determined for each condition, using real-time fluorescence detection as described above. Buffer optimization experiments were conducted at 37 °C. For the temperature optimization, the well plate was incubated at 30-55°C with a 5 °C step-size (Figure S8). Buffer optimization experiments were previously presented in van Dongen, J.E. (2022).^45^

#### Michaelis Menten kinetics

For Michaelis Menten analysis, reporter concentrations were varied, while keeping RNP and activator levels constant at 0.5 nM and 8 nM respectively. The maximal slope was determined in MATLAB (R2023b, The MathWorks, Natick, MA, USA), using a sliding window size width of 5 data points (12 minutes) to compensate for warm-up effects (Figure S9). The slope at each reporter concentration was then fit to the Michaelis Menten equation, resulting in V_max_ (horizontal asymptote) and K_M_ (the reporter concentration ½·V_max_) estimates. Calibration experiments (Figure S10), similar to as described by Ramachandran et al.,^27^ were run to convert fluorescent units to reporter concentrations.

#### Microfluidic chip fabrication

The chip layout was designed using CleWin (WieWeb software), making use of the integrated MATLAB API. Chips were fabricated using soft lithography. Using standard cleanroom techniques, a silicon wafer with a 15 μM SU-8 layer was produced to be used as a mold. Polydimethylsiloxane (PDMS, SYLGARD™ 184 silicone elastomer) was mixed with curing agent (10 : 1 w/w), degassed, poured onto the mold, and baked at 60 °C for at least three hours. Afterwards, the PDMS was peeled off the mold and cut into chips. The chips were bonded to standard glass microscopy coverslips by contact after 60 seconds of O2 plasma cleaning (model CUTE, Femto Science, Hwaseong-Si, South Korea), followed by another 20-minute bake at 60 °C. In preparation for droplet generation, the chip was again plasma-cleaned, after which Aquapel® (PPG Industries, Inc., Pittsburgh, PA, USA) was inserted into the oil inlet using a syringe with a blunt tip needle to make the oil channel hydrophobic. Lastly, the Aquapel® was removed again by applying compressed air to the sample inlet, and baking the chip on a hotplate at 100 °C for one hour.

#### Digital droplet assay

Droplets were generated by filling the chip from the back inlet using FluoSurf droplet generation oil (Emulseo, France) and pressure-driven pumps (LineUp™ series, Fluigent) to displace air. Oil was reconnected to the continuous phase inlet, and the CRISPRdx mix was emulsified via the disperse phase inlet. Tubing was connected to the back inlet and inserted into 0.2 mL with a small layer of mineral oil to collect oil waste. Inlet pressures were adjusted until stable monodisperse droplets of the desired radius were formed. After droplet generation, inlet pressure was reduced to a minimum, and pressure equilibrium was maintained by connecting a mineral oil reservoir during incubation on a hotplate at 44°C for 2 hours. Droplet fluorescence was imaged post-incubation.

#### Statistical modelling

For our statistical modeling, we implemented the equations presented by Majumdar et al.^46^ in MATLAB, adapting the model to solve for the limit of detection (LoD) with our parameter ranges of interest.

#### Data processing

Fluorescence images were processed through a series of MATLAB scripts that filter out a collection of droplets with comparable size and analyze their fluorescence (Figure S11). To count the number of targets in the fluorescent droplets, droplet fluorescence was quantified using a metric to differentiate fluorescent droplets in the distribution. As a metric, mean droplet fluorescence was divided by the median fluorescence of neighboring droplets. Neighboring droplets were defined as ≥4, but ≤11, droplets within a radius of 32.6 μm, with +10.9 μm radial increments when no droplets were found. Further data processing for figures was performed using a combination of Microsoft Excel (Microsoft Corporation, Redmond, WA, USA), MATLAB plotting functions, OriginPro 2023 (OriginLab Corporation, Northampton, MA, USA), and Graphpad Prism (GraphPad Software, USA).

### Quantification and statistical analysis

Statistical analysis for Figure 1D was performed using Prism 10 (GraphPad). The 2-way ANOVA was conducted to determine the effect of dual multiplexed detection. Significance is denoted as follows: ∗ = *p* ≤ 0.05; ∗∗ = *p* ≤ 0.01; ∗∗∗ = *p* ≤ 0.001; ∗∗∗∗ = *p* ≤ 0.0001.
