# Supplementary material for: Toward point-of-care and amplification-free detection of human cytomegalovirus using CRISPR-Cas12a
Source: iScience. 2026 Jun 23;29(7):116494. doi: 10.1016/j.isci.2026.116494 (PMC13320335; doi:10.1016/j.isci.2026.116494)
Supplement: Document S1. Figures S1–S14 and Tables S1–S5 [file mmc1.pdf]

iScience, Volume *29*

Supplemental information

Toward point-of-care and amplification-free detection of human cytomegalovirus using CRISPR-Cas12a

Kavish A.V. Kohabir, A.W.J. Rietveld, Lars O. Nooi, Roos E. Beijer, Jeanne E. van Dongen, Jasper Linthorst, Rob M.F. Wolthuis, Marcel Jonges, Matthijs R.A. Welkers, Loes I. Segerink, and Erik A. Sistermans

Supplemental Information

Supplemental Tables

Supplemental Table S1. Oligonucleotides used in this study.
Supplemental Table S2. Plate reader settings.
Supplemental Table S3. Reaction buffers used in this study.
Supplemental Table S4. Values used for kinetics Back-of-the-Envelope calculations.
Supplemental Table S5. Resulting values for kinetics Back-of-the-Envelope calculations.

Supplemental Figures

Supplemental Figure S1. Sensitivity is not influenced by doubling Cas12a concentration nor ribonucleoprotein pre-formation for both UL54 and UL55 detection.
Supplemental Figure S2. Supplemental Figure S2. Dual-multiplexed detection in clinical isolates with PCR pre-amplification.
Supplemental Figure S3. Percentual increase with each additional multiplexed crRNA.
Supplemental Figure S4. AsCas12a collateral activity has a preference for cytosine-rich ssDNA reporters.
Supplemental Figure S5. Reporter length shows a trade-off between fluorescence and background.
Supplemental Figure S6. Response using alternative reporters at various in-reaction target (UL100) concentrations.
Supplemental Figure S7. Optimization of the reaction buffer of the Cas12a assay.
Supplemental Figure S8. Optimization of the temperature of the Cas12a assay.
Supplemental Figure S9. Source data of the Michaelis Menten for the optimized assay.
Supplemental Figure S10. Calibration curves used to convert from a.u. to cleaved reporter concentration.
Supplemental Figure S11. Series of screen captures showing the analysis workflow.
Supplemental Figure S12. Droplet radius distribution change over the incubation period.
Supplemental Figure S13. Statistical modeling suggests a combination of smaller droplets and multiplexing for sensing clinical concentrations.
Supplemental Figure S14. Distribution of fluorescence in shrunken droplets suggests more favourable kinetics with smaller radius

Supplemental Tables

Supplemental Table S1. Oligonucleotides used in this study. *All oligonucleotides were ordered from Integrated DNA Technologies (IDT). Coding of chemical modifications and/or proprietary groups are as provided by the manufacturer. Annealing parts of crRNAs and relevant mutations are underlined and indicated in red respectively.*

No.	Name	Type	Sequence (5'->3')	Length
**crRNAs**				
KR158	UL54 CMV LbCas12a crRNA	crRNA	/AltR1/rUrArArUrUrUrCrUrArCrUrArArGrUrGrUrArGrArUrCrCrCrCrCrGrUrCrUrCrGrUrArGrCrArCrArCrGrC/AltR2/	42 nt
KR175	UL55 CMV LbCas12a crRNA	crRNA	/AltR1/rUrArArUrUrUrCrUrArCrUrArArGrUrGrUrArGrArUrArArGrUrGrUrCrGrGrUrArGrCrCrGrUrUrUrUrG/AltR2/	42 nt
KR237	UL54 CMV crRNA AsCas12a	crRNA	/AltR1/rUrArArUrUrUrCrUrArCrUrCrUrUrGrUrArGrArUrCrCrCrCrCrGrUrCrUrCrGrUrArGrCrArCrArCrGrC/AltR2/	41 nt
KR238	UL55 CMV crRNA AsCas12a	crRNA	/AltR1/rUrArArUrUrUrCrUrArCrUrCrUrUrGrUrArGrArUrArArGrUrGrUrCrGrGrUrArGrCrCrGrUrUrUrUrG/AltR2/	41 nt
KR264	UL10 CMV crRNA AsCas12a	crRNA	/AltR1/rUrArArUrUrUrCrUrArCrUrCrUrUrGrUrArGrArUrGrGrArGrGrGrUrCrGrUrArGrGrUrGrCrArGrArUrA/AltR2/	41 nt
KR265	UL13-UL14 CMV crRNA AsCas12a	crRNA	/AltR1/rUrArArUrUrUrCrUrArCrUrCrUrUrGrUrArGrArUrGrArArUrArCrArCrCrGrArGrCrArUrUrCrCrUrU/AltR2/	41 nt
KR266	UL25 CMV crRNA AsCas12a	crRNA	/AltR1/rUrArArUrUrUrCrUrArCrUrCrUrUrGrUrArGrArUrGrUrCrCrCrArCrCrArUrCrArGrCrArArCrArGrUrA/AltR2/	41 nt
KR267	UL48 CMV crRNA AsCas12a	crRNA	/AltR1/rUrArArUrUrUrCrUrArCrUrCrUrUrGrUrArGrArUrGrGrUrCrCrArCrArArCrUrCrCrUrGrArUrCrCrArU/AltR2/	41 nt
KR268	UL74 CMV crRNA AsCas12a	crRNA	/AltR1/rUrArArUrUrUrCrUrArCrUrCrUrUrGrUrArGrArUrArUrGrUrArCrCrCrCrArGrUrCrArGrCrArArArArA/AltR2/	41 nt
KR269	UL75 CMV crRNA AsCas12a	crRNA	/AltR1/rUrArArUrUrUrCrUrArCrUrCrUrUrGrUrArGrArUrCrCrUrGrArCrGrArCrCrGrUrGrCrUrGrUrUrArCrG/AltR2/	41 nt
KR270	UL100 CMV crRNA AsCas12a	crRNA	/AltR1/rUrArArUrUrUrCrUrArCrUrCrUrUrGrUrArGrArUrGrCrCrGrUrGrArCrCrArUrCrUrArCrUrArCrCrUrG/AltR2/	41 nt
KR271	UL103 CMV crRNA AsCas12a	crRNA	/AltR1/rUrArArUrUrUrCrUrArCrUrCrUrUrGrUrArGrArUrCrCrCrUrCrArCrCrCrCrCrCrArArGrCrUrGrCrCrA/AltR2/	41 nt
**Complementary ssDNA oligonucleotides**				
KD163	CMV target sequence (UL54) fw	ssDNA	tgatccacggcggtgtttccccccgtctcgtagcacacgcacgagatctgaatga	55 nt
KD164	CMV target sequence (UL54) rv	ssDNA	tcattcagatctcgtgcgtgtgctacgagacggggggaaacaccgccgtggatca	55 nt
KD177	CMV target sequence 2 (UL55) fw	ssDNA	ttcttcgtcagagtctttcaagtgtcggtagccgttttttgcgatgtcgcagtcgg	55 nt

KD178	CMV target sequence 2 (UL55) rv	ssDNA	ccgactgcgacatcgcaaaaacggctaccgacacttgaaagactctgacgaagaa	55 nt
KD272	CMV UL10 target sequence fw	ssDNA	cgaaaatacgcatcctatctgcacctacgaccctcctaaacctggtagacaaaag	55 nt
KD273	CMV UL10 target sequence rv	ssDNA	cttttgtctaccaggtttaggagggtcgtaggtgcagataggatgcgtattttcg	55 nt
KD274	CMV UL13/14 target sequence fw	ssDNA	gacggcccatgacgatttagaatacaccgagccattcctttatttccccccatcc	55 nt
KD275	CMV UL13/14 target sequence rv	ssDNA	ggatgggggaaataaaggaatggctcggtgtattctaaatcgtcatgggccgtc	55 nt
KD276	CMV UL25 target sequence fw	ssDNA	cgcgacagcggggaatactgttgctgatggtgggacgaaacagcagggcggaaca	55 nt
KD277	CMV UL25 target sequence rv	ssDNA	tgttccgccctgctgtttcgtcccaccatcagcaacagtattccccgctgtcgcg	55 nt
KD278	CMV UL48 target sequence fw	ssDNA	ccacttggaccaaattttgggtccacaactcctgatccatgagacgcagcaggcc	55 nt
KD279	CMV UL48 target sequence rv	ssDNA	ggcctgctgcgtctcatggatcaggagttgtggacccaaaatttggtccaagtgg	55 nt
KD280	CMV UL74 target sequence fw	ssDNA	aacgatggcatgaaatttttgctgactggggtacattaaaaaccggtatccgtca	55 nt
KD281	CMV UL74 target sequence rv	ssDNA	tgacggataccggtttttaatgtaccccagtcagcaaaaatttcatgccatcgtt	55 nt
KD282	CMV UL75 target sequence fw	ssDNA	tgaaactgatggcgttttccctgacgaccgtgctgttacggaggctgctgttgta	55 nt
KD283	CMV UL75 target sequence rv	ssDNA	tacaacagcagcctccgtaacagcacggtcgtcagggaaaacgccatcagtttca	55 nt
KD284	CMV UL100 target sequence fw	ssDNA	cttgatccagcataccaggtagtagatggtcacggctaaaaagacgagctgcatg	55 nt
KD285	CMV UL100 target sequence rv	ssDNA	catgcagctcgtctttttagccgtgaccatctactacctggtatgctggatcaag	55 nt
KD286	CMV UL103 target sequence fw	ssDNA	ccgttcccagcgcggtggcagcttgggggtgagggcaaattggggtagttggcg	55 nt
KD287	CMV UL103 target sequence rv	ssDNA	cgccaactaccccaatttgccctcacccccaagctgccaccgcgctgggaacgg	55 nt
**PCR primers**				
KD183	CMV UL55 fw	ssDNA	agacgttctcttcttcgtcagagtc	25 nt
KD187	CMV UL55 rv	ssDNA	acctactagaccgactgcgacatcg	25 nt
KD193	CMV UL54 fw	ssDNA	ccgtttgggatcccctgatccacgg	25 nt

KD196	CMV UL54 rv	ssDNA	agtccgatgacattgtcattcagatctcgt	30 nt
**dsDNA fragments (gBlock®)**				
KD181	CMV UL54+UL55 target Gblock	dsDNA	gcttacgaagcaaaatcacaaagaaaatacacatgcag cacctagatatccagtttaaccccgtatatcacaagtc tctgtgtcacttttttttttgtctgttttttttttttcttc tcctggttcagacgttctcttcttcgtcagagtctttc aagtgtcggtagccgtttttgcgatgtcgcagtcggtc tagtaggttgggcttctgtcccttgtcctgcgtgccag tccgtccgtccaaagaatctgtgcccgcaggtgccgat cgtgaagagatgaagacccgagtgcccaaagatcacac cctccgaagtgcagccccggccatcgttcccgtttggg atcccctgatccacggcggtgtttccccccgtctcgta gcacacgcacgagatctgaatgacaatgtcatcggact tctcggcgcagggaaaccaccctcgccgctcatgcac tcgatatcgaaggacaggcatcgatagcgcggccacga gctgtc	500 nt
**Reporters**				
KD035	TTATT probe	ssDNA	/56-FAM/TTATT/3IABkFQ/	5 nt
KD220	TTATT LFA probe	ssDNA	/56-FAM/TTATT/3Bio/	5 nt
KD288	5A FAM probe	ssDNA	/56-FAM/AAAAA/3IABkFQ/	5 nt
KD289	5C FAM probe	ssDNA	/56-FAM/CCCCC/3IABkFQ/	5 nt
KD290	5G FAM probe	ssDNA	/56-FAM/GGGGG/3IABkFQ/	5 nt
KD291	5T FAM probe	ssDNA	/56-FAM/TTTTT/3IABkFQ/	5 nt
KD293	6C FAM probe	ssDNA	/56-FAM/CCCCCC/3IABkFQ/	6 nt
KD294	7C FAM probe	ssDNA	/56-FAM/CCCCCCC/3IABkFQ/	7 nt
KD295	8C FAM probe	ssDNA	/56-FAM/CCCCCCCC/3IABkFQ/	8 nt
KD296	9C FAM probe	ssDNA	/56-FAM/CCCCCCCCC/3IABkFQ/	9 nt
KD297	10C FAM probe	ssDNA	/56-FAM/CCCCCCCCCC/3IABkFQ/	10 nt
KD298	11C FAM probe	ssDNA	/56-FAM/CCCCCCCCCCC/3IABkFQ/	11 nt
KD299	12C FAM probe	ssDNA	/56-FAM/CCCCCCCCCCCC/3IABkFQ/	12 nt
Stem-loop #10T	10T hairpin FAM probe	ssDNA	/56-FAM/CTCTCATTTTTTTTTTAGAGAG /3IABkFQ/	22 nt
TTATT-5C	TTATT-5C FAM probe	ssDNA	/56-FAM/TTATTCCCCC/3IABkFQ/	10 nt

Supplemental Table S2. Plate reader settings. *For kinetic fluorescence monitoring, we used the following settings on the Infinite® 200 Pro M Plex plate reader (Tecan Group Ltd.).*

**Mode**	**Fluorescence top reading**
Temperature	37 °C
Excitation	485 nm
Emission	535 nm
Excitation bandwidth	9 nm
Emission bandwidth	20 nm
Gain	85 (manual)
Number of flashes	10
Integration time	20 µs
Lag time	0 µs
Settle time	0 µs
Z-position (manual)	16814 µM

Supplemental Table S3. Reaction buffers used in this study.

	Tris HCl	MgCl2	NaCl	DTT	PEG-200	pH
Buffer-Na^+	10 mM	10 mM	-	1 mM	-	8
Buffer-Mg^{2+}	10 mM	-	10 mM	1 mM	-	8
Buffer-DTT	10 mM	10 mM	-	-	-	8
Buffer-Tris-HCl	-	15 mM	-	-	-	-
Buffer-PEG	10 mM	15 mM	-	-	-	8
Optimized Buffer	10 mM	15 mM	-	2 mM	2.50% (V/V)	8.5
NEB2.1	10 mM	10 mM	50 mM	-	-	8

Supplemental Table S4. Values used for kinetics Back-of-the-Envelope calculations.

Activator	K_{cat} (1/s)	K_M (M)	K_{cat} / K_M (1/Ms)	v (nM/s)	t_{lin} (s)	S_0 (nM)	E_0 (nM)
A.s.Cas12a Ultra	2.17	1.76×10^{-6}	1.23×10^{6}	0.42*	1800	1000	0.5

*This is half the reported value in the Michaelis Menten plot, as this activity is normalized to E_0 = 1 nM

Supplemental Table S5. Resulting values for kinetics Back-of-the-Envelope calculations

Activator	K_{cat} (1/s)	K_M (M)	K_{cat} / K_M (1/Ms)	α	β	γ	Checks Violated
A.s.Cas12a Ultra	2.16	1.76×10^{-6}	1.23×10^{6}	0.756	0.19	1.11	None

Supplemental Figures

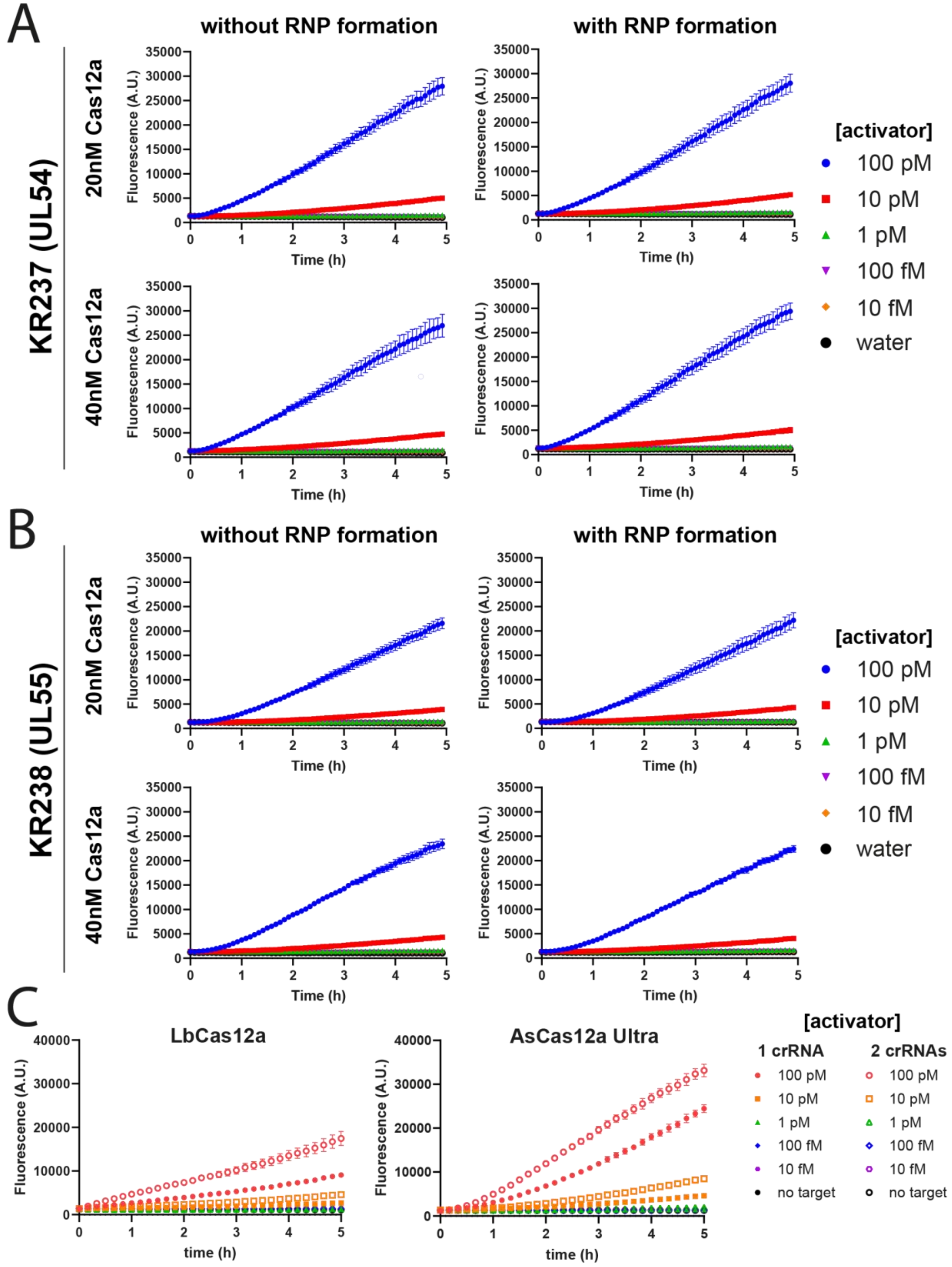

(caption on next page)

Supplemental Figure S1. *Sensitivity is not influenced by doubling Cas12a concentration nor ribonucleoprotein pre-formation for both (A) UL54 and (B) UL55 detection. (C) LbCas12a has slower reaction kinetics and compared to AsCas12a Ultra, irrespective of dual-multiplexed detection. Graphs depict mean fluorescence values of triplicate reactions, with error bars indicating standard deviation of the mean. All denoted molarities represent in-reaction concentrations.*

A

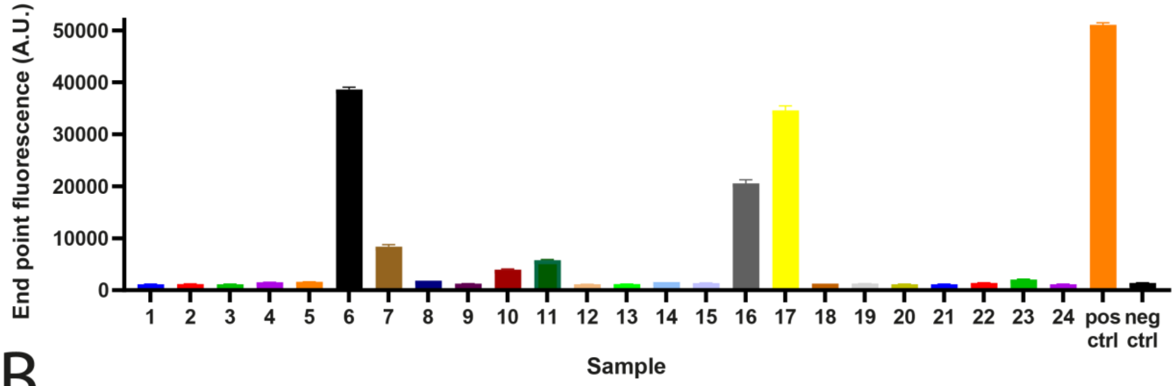

B

Sample	1	2	3	4	5	6	7	8	9	10	11	12
cp/mL	0,0	0,0	0,0	248	748	410084	33556	2724	0	2724	7156	0,0
aM	0,0	0,0	0,0	0,4	1,2	681,0	55,7	4,5	0,0	4,5	11,9	0,0

Sample	13	14	15	16	17	18	19	20	21	22	23	24
cp/mL	0,0	0,0	0,0	90668	138104	0	0	248	0,0	0,0	0,0	0,0
aM	0,0	0,0	0,0	150,6	229,3	0,0	0,0	0,4	0,0	0,0	0,0	0,0

Supplemental Figure S2. Dual-multiplexed detection in clinical isolates with PCR pre-amplification. *(A) Fluorescence end-point values after 1h incubation. Error bars display standard deviation of triplicate reactions. (B) Corresponding viral loads as determined by qPCR. A sample concentration of 500cp/mL (0,83aM) corresponds to adding 1 copy to the PCR reaction.*

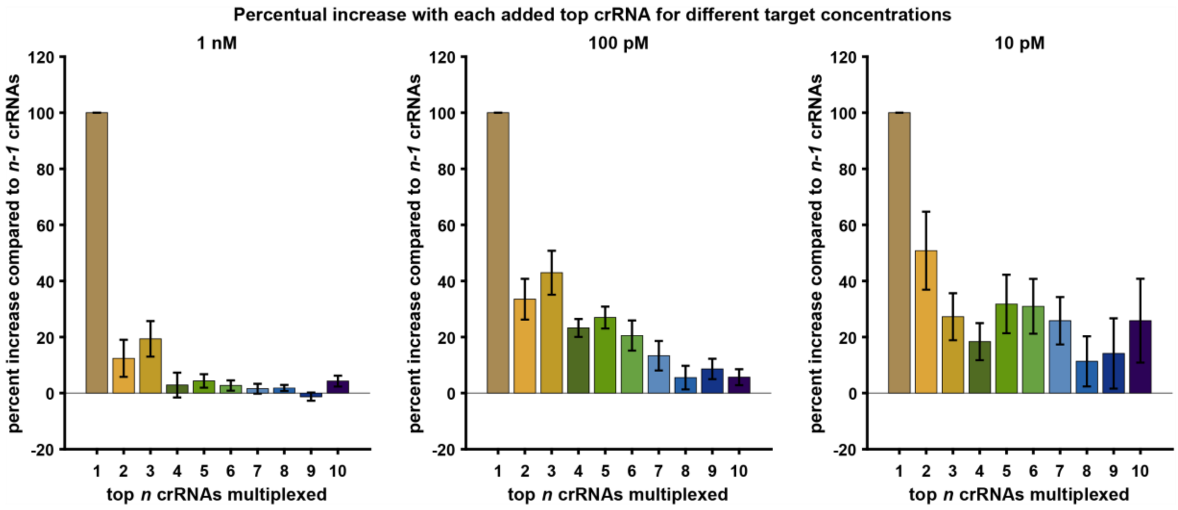

Supplemental Figure S3. Percentual increase with each additional multiplexed crRNA. Based on results of multiplexed detection after 5 hours of incubation when using 100 pM, 10 pM or 1 pM of in-reaction activator cocktails. Error bars indicate the standard error of the mean of triplicate reactions. crRNA cocktails were kept at 25 nM total RNA in-reaction concentration. All used activator cocktails consist of equimolar amounts of all 10 targets, and the reported concentrations represent the level of each target.

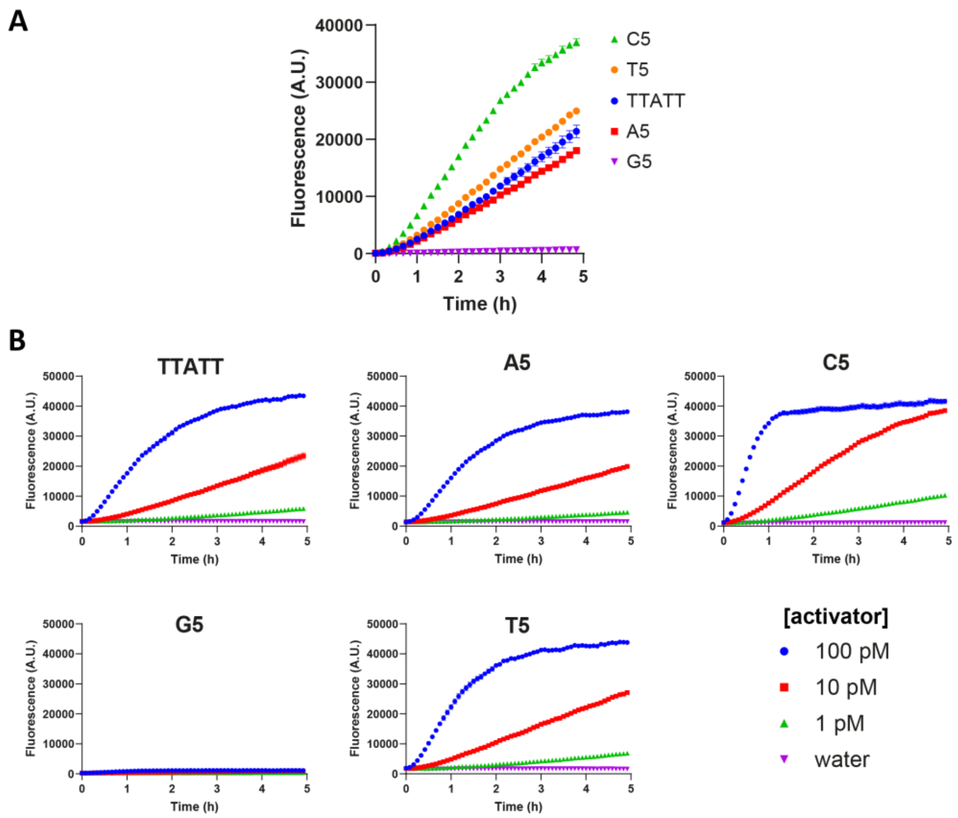

Supplemental Figure S4. AsCas12a collateral activity has a preference for cytosine-rich ssDNA reporters. *(A) Background subtracted fluorescence curves for 10-multiplexed detection of 10pM in-reaction activator cocktail. (B) fluorescence data for the individual reporters at different concentrations*

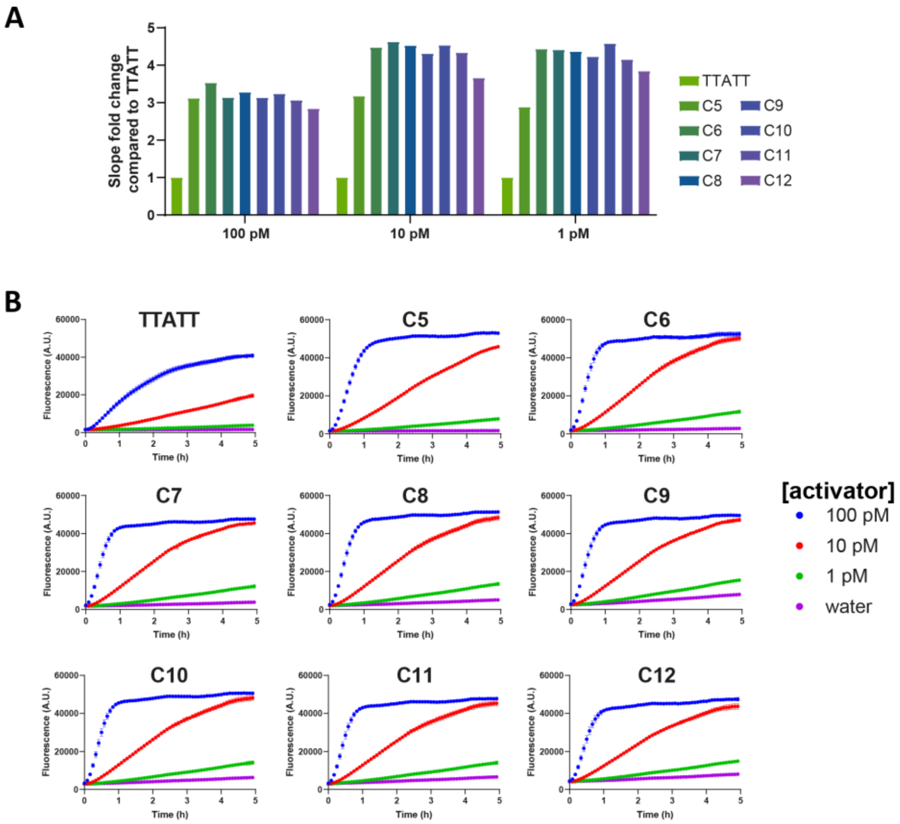

Supplemental Figure S5. Reporter length shows a trade-off between fluorescence and background. *(A) Fluorescence slope fold changes using cytosine-rich probes compared to the TTATT probe. Slopes were calculated with linear regression on datapoints within the first hour of incubation. All graphs depict mean values of triplicate reactions, with error bars displaying the standard error of the mean. (B) Raw fluorescence data comparing reporter length up to 12 nucleotides. Graphs display mean fluorescent values of triplicate reactions, with error bars displaying the standard deviation of the mean.*

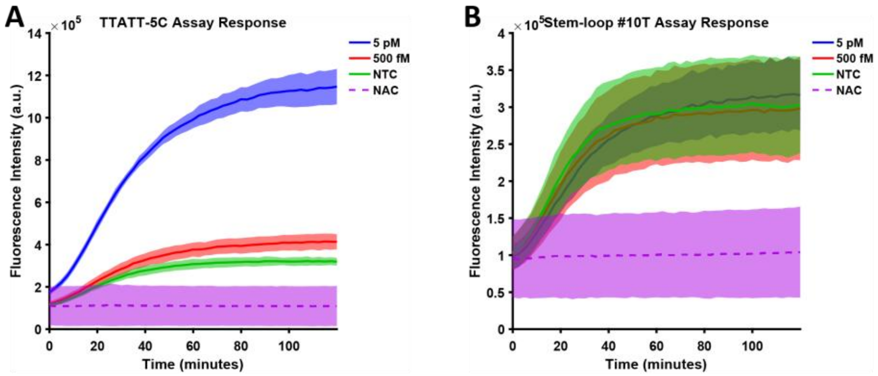

Supplemental Figure S6. Response using alternative reporters at various in-reaction target (UL100) concentrations. *(A) Raw fluorescence data showing the response of a TTATT-5C reporter. (B) Raw fluorescence data showing the response of a hairpin reporter (stem-loop #10T)*

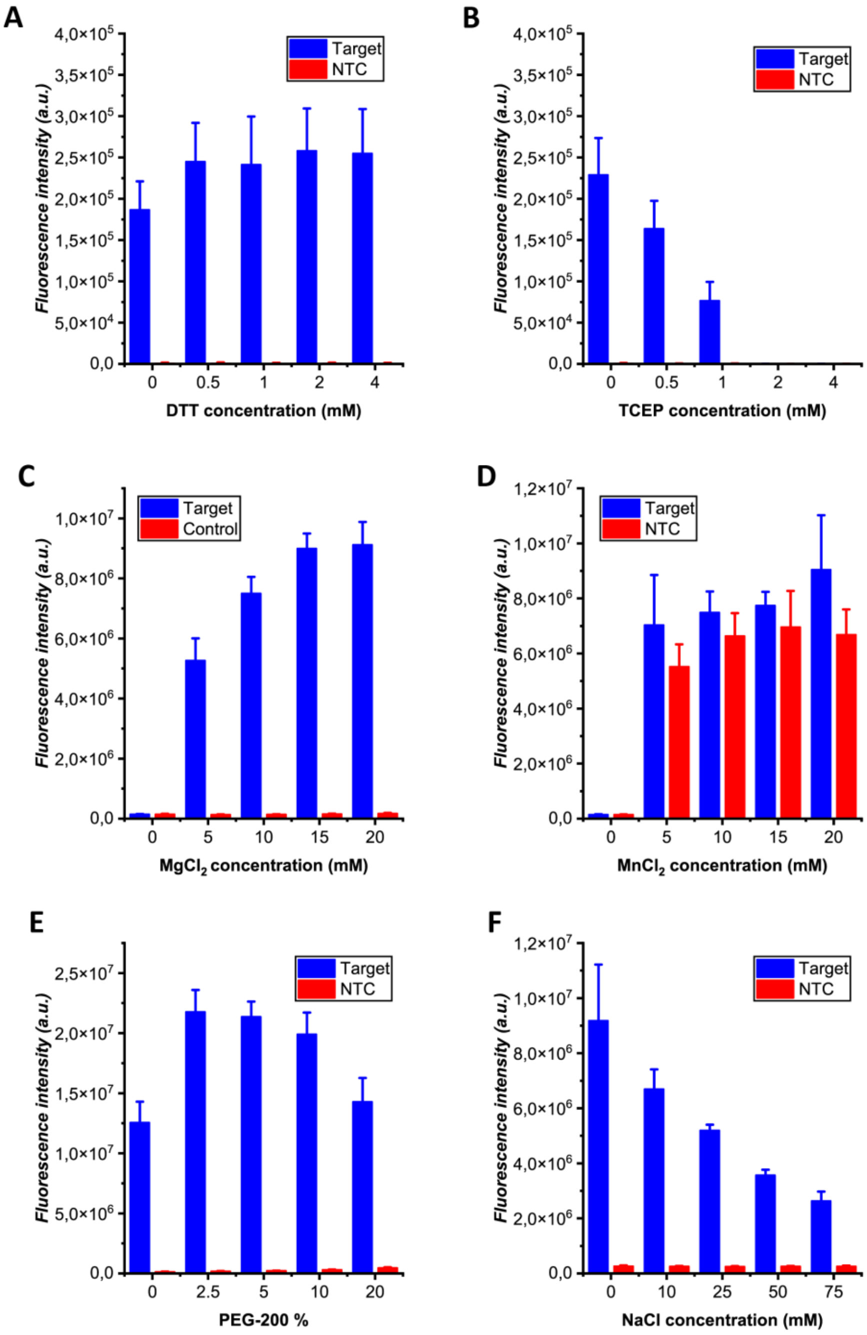

Supplemental Figure S7. Optimization of the reaction buffer of the Cas12a assay. *(A) Effect of the DTT concentration (B) Effect of the TCEP concentration (C) Effect of the $MgCl_2$ concentration (D) Effect of the $MnCl_2$ concentration (E) Effect of the PEG-200 % (F) Effect of the NaCl concentration*

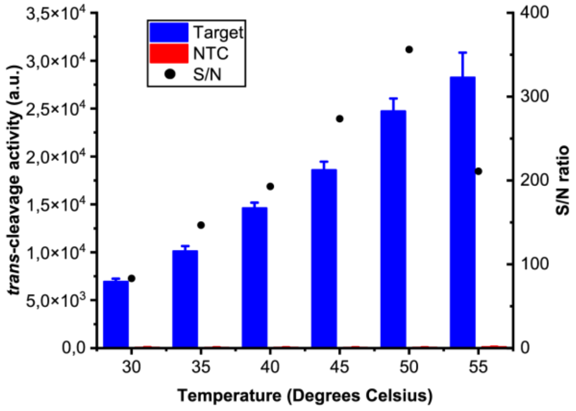

Supplemental Figure S8. Optimization of the temperature of the Cas12a assay. *Data are represented as mean ± standard error of the difference between means of three absolute and three technical replicates (n=9). Black circles represent the signal/noise ratio.*

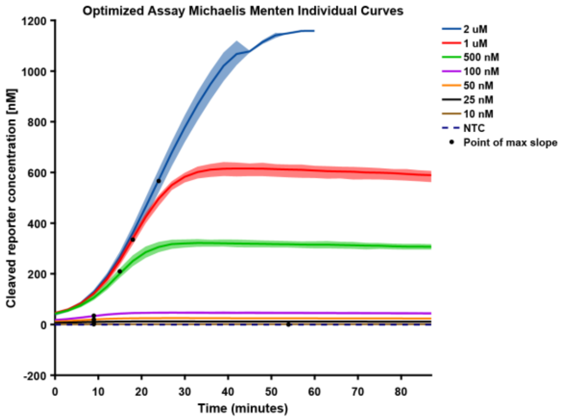

Supplemental Figure S9. Source data of the Michaelis Menten for the optimized assay. *Shaded area shows the standard error of the mean of two absolute and three technical replicates. The black circles shows the points at which the maximum slope is reached (using a sliding window of width five), which is used to compensate for warm-up effects.*

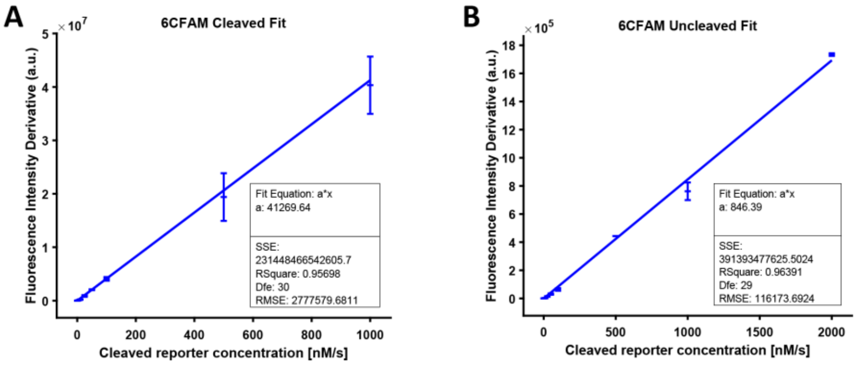

Supplemental Figure S10. Calibration curves used to convert from a.u. to cleaved reporter concentration. *Error bars show the mean ± standard error of the means of two absolutes with each three technical replicates.*

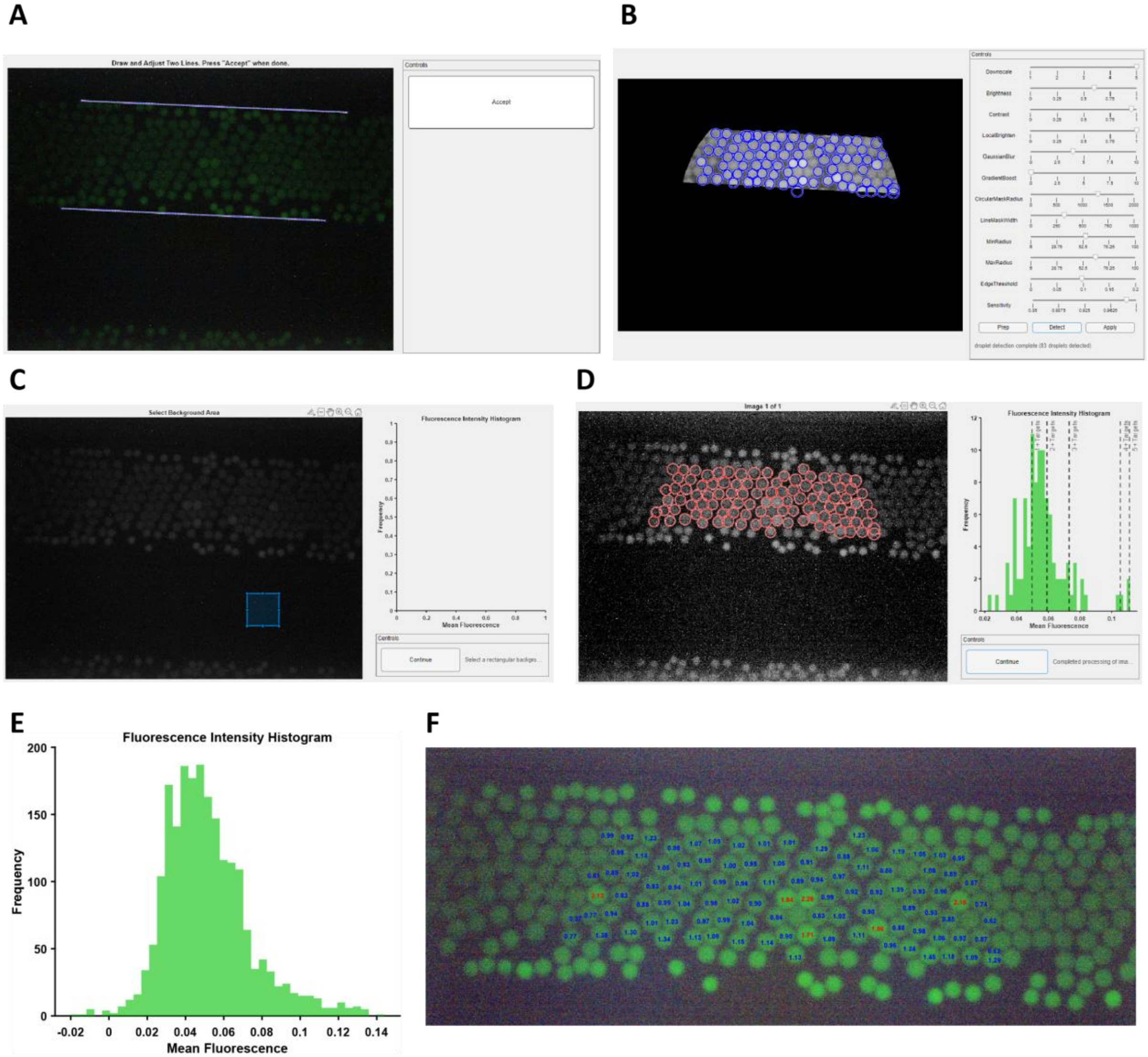

Supplemental Figure S11. Series of screen captures showing the analysis workflow. *For each image, (A) the user draws two lines on each image at the sides of the channel (B) Using the line data, a part of the image is masked off. Images are pre-processed to enhance circle detection. (C) The user specifies an area for background subtraction (D) The raw fluorescence data is analyzed for each image, and the detected circles are showed to the user for validation. (E) the resulting raw fluorescence distribution (N=2071). (F) Output of the metric calculation placed overtop a representative image. Red text shows those droplets that have a score higher than the chosen threshold of 1.5.*

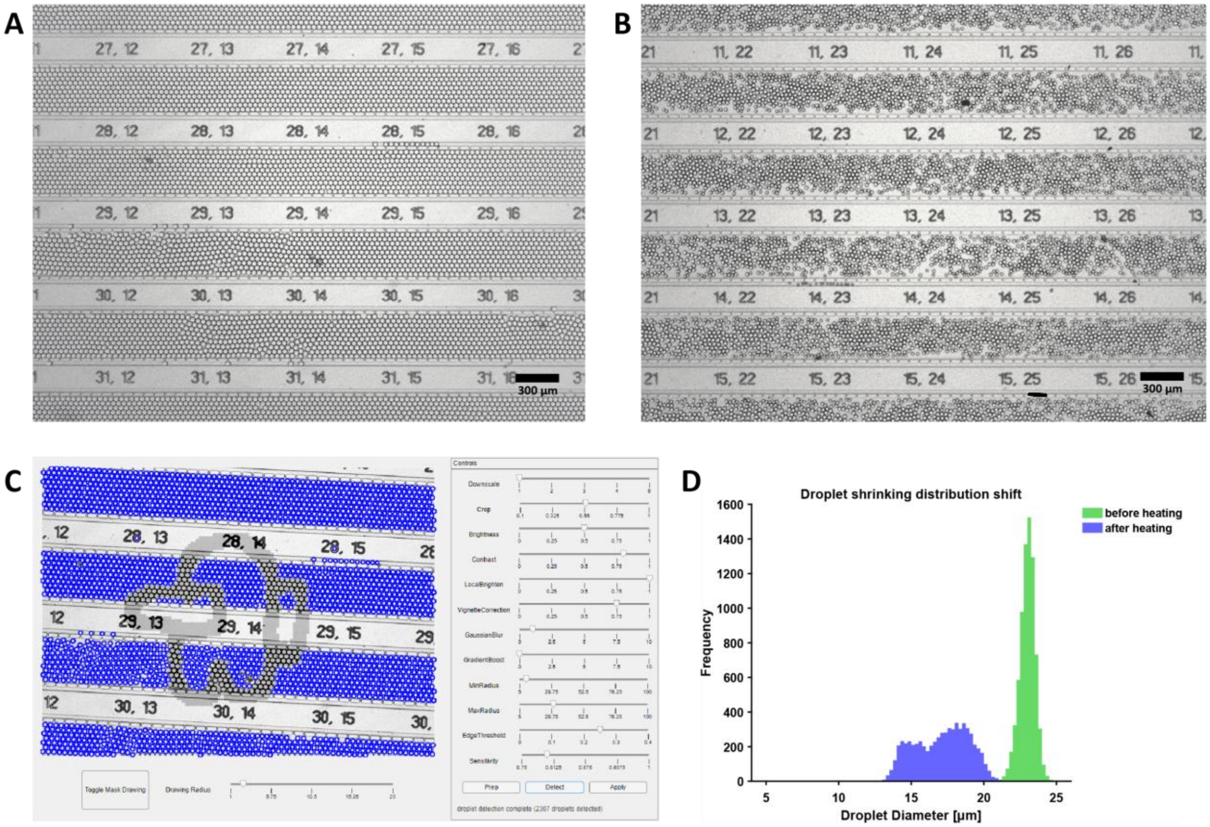

Supplemental Figure S12. Droplet radius distribution change over the incubation period. *(A) microscope image of the droplets before incubation. (B) A microscope image of the droplets after incubation where the droplets have shrunk. (C) Image showing a screen capture of the radius analysis script. In a GUI, sliders are used to control the pre-processing and droplet detection settings. A mask can be drawn by hand to prevent detection in areas outside the channel, such as on the positional markings. For demonstration purposes, here a mask is drawn overtop the channels, showing how droplets are not detected in the masked area. (D) Histogram output of the analysis script for the droplet distribution before (Volume: 5353.14 ± 278.79 fl, Diameter: 23.00 ±0.52 µm, N=7808), and after heating (Volume: 2603.22 ±741.94 fl, Diameter: 17.10 ±1.84 µm, N=7332).*

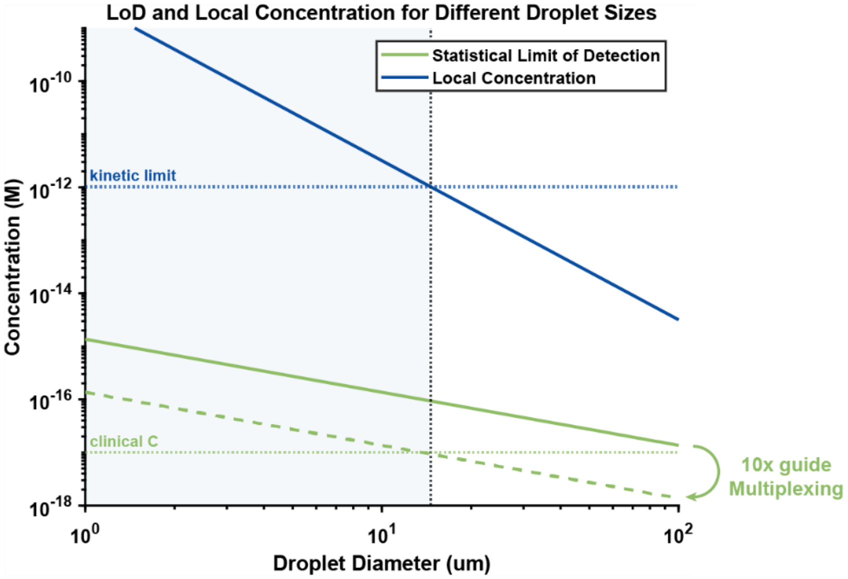

Supplemental Figure S13. Statistical modeling suggests a combination of smaller droplets and multiplexing for sensing clinical concentrations. *While we saw fluorescence in droplets without amplification, quantification of low concentration samples is not possible without single molecule detection. Using the assay results, we can evaluate under what conditions quantitative analysis of hCMV samples using microfluidic techniques is feasible. Majumdar et al. derived equations on how the parameters of a digital assay relate to its attainable LoD [25]. For our study, we use a droplet count corresponding to a fixed chip area of 6 cm2 and set requirements on the precision and confidence level to 10% and 90%, respectively*

Relation between droplet radius, local concentration and achievable LoD. The blue line shows the local target molecule concentration for different radii. In our digital droplet assay, signal was detectable for droplets with a local concentration above 12 pM, labeled as the kinetic limit (shaded area). The green line shows the LoD as modelled for different radii in a chip with a fixed area of 6 cm^2. This shows how the low attomolar LoD of clinical samples is not reached with droplet radii where the kinetics are sufficient. Multiplexing could serve to decrease the LoD closer to the clinical limit (green dashed line).

Reducing droplet size increases local target concentration, reaching the detection limit seen in the assay at diameters around 10 µm. However, it follows from the model that achieving aM sensitivity requires further turnover optimization. Multiplexing strategies can be modelled as an effective increase of the number of detectable fragments. To illustrate this, we plot how 10x guide multiplexing would lower the LoD , showing how low aM concentrations could then be reached at droplet radii where the local target concentration is also sufficient.

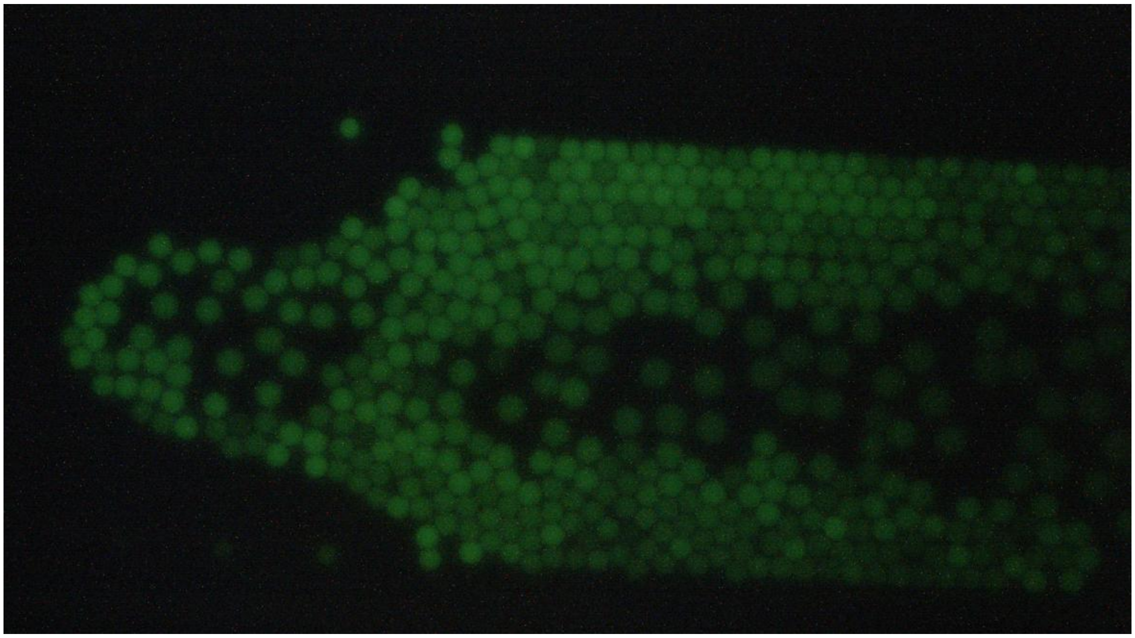

Supplemental Figure S14. Distribution of fluorescence in shrunken droplets suggests more favourable kinetics with smaller radius. *Figure depicts results in an experiment where in a region of the board the droplets shrunk homogeneously to a small diameter of 13.1±0.1 μm, corresponding to a local concentration of 51pM. This experiment used a sample concentration containing 4 pM of target and initial droplet sizes of 23 μm diameter, so that 73 % of droplets is expected to have at least one target.*

The brightness distribution appears more comparable to the mix expected for a digital result, suggesting that the signal is enhanced by shrinking. An increase in background is also expected with droplet shrinking, which may explain why the contrast remains limited. Unfortunately, the low droplet count and contrast make the single image unsuitable for rigorous analysis.
